# Psychological safety during the test of new work processes in an emergency department

**DOI:** 10.1186/s12913-022-07687-y

**Published:** 2022-03-05

**Authors:** Peter Dieckmann, Simon Tulloch, Anne Eva Dalgaard, Kirsten Varming

**Affiliations:** 1 Copenhagen Academy for Medical and Educational Simulation (CAMES), Center for Human Resources and Education, Herlev Hospital, 25 Thfloorfloor, Borgmester Ib Juuls Vej 1, 2730 Herlev, Capital Region Denmark; 2 Department of Public Health, University of Copenhagen, Copenhagen, Denmark; 3 Department of Quality and Health Technology, University of Stavanger, Stavanger, Norway; 4 Danish Society for Patient Safety, Frederiksberg, Denmark; 5 Herlev and Gentofte Hospital, Herlev, Capital Region Denmark

**Keywords:** Emergency medicine, Psychological safety, Improvement, Context

## Abstract

**Background:**

Emergency medicine is a complex setting for healthcare delivery which relies on communication, negotiation, teamwork, trust, and shared dialog. The nature of the work comprises dealing with emotionally challenging situations and acting under uncertainty. For healthcare staff this poses the need to be adaptive and open to change. Psychological safety is an important component of productive teamwork and learning in such contexts. Edmondson’s model of team psychological safety highlights factors which contribute to the development of psychological safety for staff groups and the mediating role this has for team performance.

**Aim:**

The aim of the study was explore the link between psychological safety and improvement work. The research question was: Do the aspects covered in the Edmondson model fully describe healthcare workers’ perceptions of psychological safety and are all aspects in the model needed to describe these perceptions during testing of new work procedures in an emergency department?”

**Methods:**

Using a mixed-method approach we investigated a change programme with interviews, a questionnaire and a workshop in an emergency department of a hospital in the Capital Region of Denmark. Thematic analysis of qualitative data and descriptive statistics of questionnaire data were undertaken.

**Results:**

Data indicate the Edmondson model is useful to help understand and identify important antecedent and outcome factors during a period of testing new work-flow processes. The model could not capture all aspects in this study’s data material, and was updated as a result. The main modifications were explicitly integrating the physical aspects of the work setting into the considerations of psychological safety, the inclusion of an additional antecedent factor relating to perceptions of care quality and adopting bi-directional links between the antecedent and consequence elements in the model.

**Conclusions:**

Although limited in scale, the study supports Edmondson’s model of psychological safety as appropriate in describing many of the dynamics experienced by staff engaged in testing new work process. However, additional factors, not included in Edmondson’s model and potential adaptations to the model are proposed.

**Supplementary Information:**

The online version contains supplementary material available at 10.1186/s12913-022-07687-y.

## Background

Emergency Departments (ED) have been described as complex and adaptive work environments which rely on communication, negotiation, teamwork, trust and shared dialog [1, 2]. To ensure safe, effective, and high-quality care in such variable conditions, improvement work is part of the daily operations [3, 4]. Examples which may require improvement methodologies, could be the integration of new guidelines into existing work processes, new devices requiring changes in the patterns of collaboration, or incidents highlighting safety gaps in current practice. The actual improvement under investigation in this study relates to new treatment pathways for patients admitted to an emergency department and is described in detail in the method section under the heading of (the change under investigation). In 2016, the Danish government launched a national healthcare quality programme which formalized the requirement and established a framework for continuously improving the quality of care in the healthcare system [5]. Furthermore, the government committed to building a number of new hospitals more appropriate to the changing demands of providing healthcare in the twenty-first century [6]. This context provides an ideal opportunity to investigate improvement work.

The improvement work in this context utilizes improvement science methodology and plan-do-study-act (PDSA) cycles. PDSA cycles provide a structure for iterative testing of changes to improve the quality of systems. The method is widely accepted in healthcare improvement but originates from industry and Walter Shewhart and Edward Deming [7]. The approach is centered around three questions: What do we want to achieve? How do we know that a change is actually an improvement? Which changes should we initiate to create improvements? These changes are tested on a small scale as a means of building knowledge and confidence regarding the change idea. For the current paper, we are interested in what role psychological safety plays in this process, where we assume that there will be more initial ideas, a deeper processing of individual ideas, a more rigorous test of ideas, etc., if those involved feel psychologically safe [8].

Psychological safety describes the degree to which people perceive their work environment as being supportive of interpersonally risky behaviours [9]. Doing improvement work is rich in such risks, for example, trying a new approach that makes one look less competent than one would like to look in front of colleagues; not understanding a change in practice; disagreeing with a proposed change; or insecurity about the effects of a change on one’s work [8].

**Fig. 1:**
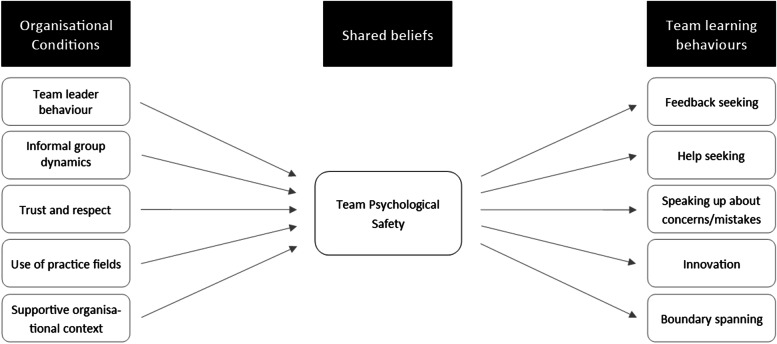
The model of psychological safety inspired by [9]

The perception of psychological safety is influenced by contextual factors which can be regarded as ‘antecedents’ (see Fig. 1 for details). Workers who feel psychologically safe are, for example, more likely to engage in activities which can broadly be described as learning behaviours [9], such as ‘asking for help’, ‘speaking up’, etc. This model builds on the underlying premise that people are (both conscious and unconscious) impression managers, i.e., reluctant to engage in behaviours that could threaten the image others hold of them [9, 10]. For Kahn, psychological safety is experienced as “feeling able to show and employ one’s self without fear of negative consequences to self-image, status, or career” [11]. Thus, improving the quality of work processes and outcomes requires effort and engagement which Kahn defines as being physically, cognitively, and/or emotionally connected to the improvement work [11]. Engagement is essential for overcoming powerful barriers to quality improvement that exist in the health care setting, as well as in other busy and chaotic service contexts [8]. Health care professionals are often stretched thin, often extremely challenged to complete their requisite tasks, let alone devote time to improving the system [12, 13]. Participating in quality improvement efforts thus requires deliberate and effortful allocation of time. Yet, despite time and resource constraints, many in health care are embracing quality improvement projects, because of what is at stake when systems fail. The construct of engagement captures the commitment and effort these individuals devote to quality improvement.

Psychological safety is critical in a volatile, uncertain, complex, and ambiguous (VUCA) world [14]. The more complex and uncertain an environment, the more important it is for people in it to feel psychologically safe so that they can engage fully in the task. In environments that fail to provide psychological safety, staff are less likely to speak up about problems they experience in everyday clinical work [14, 15] and be less engaged in their work [8]. Improvement work by definition involves change [16] and change poses extra challenges for psychological safety, as such, testing the model in such a VUCA context as an ED during a change in work processes can be seen as a rigorous test of its applicability.

In this paper we focus on ‘staff perceptions’ of contextual factors which may impact on improvement work in an ED. Specifically, we investigate whether the Edmondson model of psychological safety [9] facilitates an understanding of staff perceptions of readiness for engagement in improvement work. An improved understanding of these issues is likely to be relevant for other hospitals in and beyond Denmark that engage in testing new and improving existing work structures and processes, whether in the context of a move to a new building or otherwise.

The aim of the study was to improve our understanding of how the factors related to psychological safety are relevant in such conditions. This is useful information, as it may influence how teams in similar contexts engage in future improvement work.

Based on the theoretical assumptions and the problem description, we formulate the following rese “Do the aspects covered in the Edmondson model fully describe healthcare workers’ perceptions of psychological safety and are all aspects in the model needed to describe these perceptions during testing of new work procedures in an emergency department?” This research question explores the link between psychological safety and improvement work.

## Methods

### The setting

The study was conducted in the Emergency Department (ED) of Herlev and Gentofte Hospital in the Capital Region of Denmark. The department treats approximately 150,000 patients annually. The department employs approximately 250 staff, including the leadership team, nursing, and administrative staff. Physicians are not directly employed in the ED, but in the departments of their respective specialty (e.g., internal medicine). Each day a pre-assigned group of physicians works in the ED. Additional physicians from the different specialties can be called in, when required. An internal medicine physician and a coordinating nurse manage the patient flow throughout the department. They initially assign a patient to certain clinical staff and later coordinate the patients’ continued treatment. This necessitates considerable variation in the composition of the actual care teams, depending on patient needs or resource availability.

### The change under investigation

The improvement work, utilizing by the improvement methodology described in the introduction, involved testing new treatment pathways, by specifically allocating nurses and physicians to dedicated teams responsible for patients presenting with broad categories of clinical need, such as an ‘infection’, and treating these patients in the so called “infection pathway” (the focus of this study). The primary aim of the clinical improvement project was to improve the completion of all relevant clinical tests as promptly as possible to facilitate subsequent clinical decisions such as hospital admission. The secondary aim of the clinical improvement project was to understand better the impact of having a single shared physical space for interdisciplinary collaboration and co-ordination between physicians and nurses, who previously had separate bases. This was particularly relevant in terms of a move to a new building which the department was scheduled undertake. We do not unfold all the details of the actual improvement work here, as our focus is on the role that psychological safety plays in this improvement work.

During the test, the infection pathway was allocated a dedicated room containing four beds. The team assigned to the infection pathway on a given day took their ‘work base’ in that room during their shift. Staff members not involved in the test did not use that room but continued to use the regular rooms during their shift. The coordinating nurse allocated patients to the infection pathway or the other parts of the ED at the point of presentation to the department. The test period lasted two weeks (18.02.2019 until 03.03.2019).

### Sampling

Sampling for the improvement project was purposeful, independent of the current study, and was undertaken by the ED leadership, and comprised employees known to be interested in testing new work processes. Participants were briefed regarding the test rational and procedures. During the test period, it was necessary to replace one of the nurses in the infection pathway for personal reasons. The replacement nurse was not part of the initial briefing for the test, but was briefed separately prior to starting in the team. All the participants in the test were invited to take part in the current study, investigating their perception of psychological safety during the test. All invited, agreed to participate.

### Ethics approval and consent to participate

The leadership of the participating department approved the study and its publications. All methods were carried out in accordance with relevant guidelines and regulations. They were informed about the goal and nature of the study, as well the plans to publish the data in anonymized form. They were asked for permission to audio-record the interview, which one participant declined. They were informed about the possibility to withdraw from the study at any point in time without consequences. Participants provided verbal informed consent to participate in the study after receiving this information. We deemed the verbal consent as sufficient in the current setting, because we do not report any identifying information about the participants and because of the nature of the collegial relationship to the participants. This procedure was not reviewed by the ethics committee. Danish law exempts this type of study from a formal ethical review, as it is not considered health-science research. The National Committee on Health Research Ethics (Capital Region Branch) therefore excluded the study from a formal review (reference number: H-21036410).

### Data collection

**Fig. 2:**
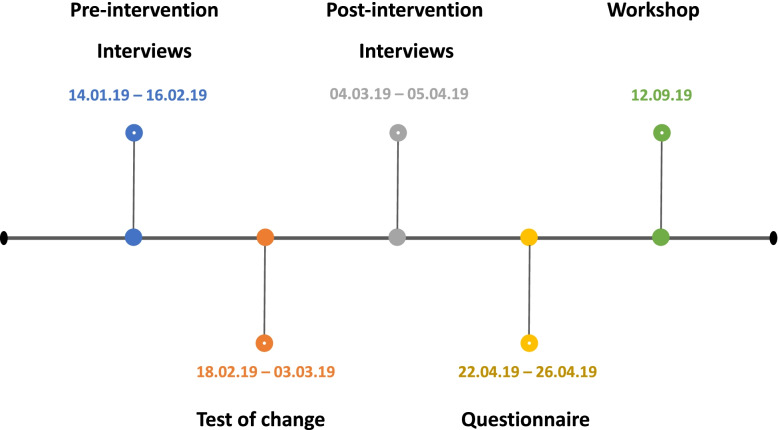
Data collection overview and timeline

The study involved three methods of data collection, administered over two phases: semi-structured interviews before and after the test period, a questionnaire [9] and a workshop after the test period. Figure 2 provides an overview of the data collection elements and their timing.

### Interviews

Participants first got the information described in the ethics section and were asked for consent for the audio recording of the interview. One participant preferred not to have the interview audio recorded, but agreed to the interviewers taking notes.

The interviews before the test aimed to identify the expectations towards the test and the post-intervention interviews to investigate their concrete experiences. The interviews lasted between 30 and 45 min. AD was present in all interviews and was accompanied by either KV or PD. One interview was conducted by AD alone. One interviewer led the interview along the interview guide, while the other occasionally asked follow-up questions. The interviews were therefore conducted in a semi-structured way. The interview guide was developed within the research team, drawing on the Edmondson model and experiences with improvement and research projects in other contexts. Interviewers independently took notes on a printed version of the interview guide. The rationale of having two interviewers present was to improve data quality with the system of note taking. The resources in the project did not allow for transcribing the interviews and therefore the extra person and set of notes was used make sure that the input by participants was accurate. This rationale was explained to participants briefly and all approved of this procedure. Notes consisted of paraphrased ‘core points’ or direct citations. The interview guide is provided as an Additional file 1. The data set consists of interview notes for all participants and audio-recordings for all but one.

### Questionnaire

A translated (Danish) version of the psychological safety questionnaire [9] was used to investigate staff perceptions of psychological safety in the team, after the post-test interviews were finished. Although this measure was developed through interviews and observations conducted within manufacturing, it is the most commonly used measure of team psychological safety and has demonstrated good psychometric properties in various settings, including healthcare [17]. The questions were provided electronically via “Mentimeter” (www.mentimeter.com), after participants gave their consent to participate. The questionnaire contains seven statements that are rated on a 7-point Likert scale (from "very inaccurate" to "very accurate."). Three items are formulated negatively. The maximum score is 49, scores above 40 indicate high levels of team psychological safety. The process is anonymous, and no descriptive personal information was collected with the questionnaire.

### Workshop

We conducted a four-hour workshop to discuss the interview and questionnaire results after the analysis of both were conducted. Of the 15 ED staff invited, 7 leaders and 2 administrative staff attended. Due to work (shift) commitments, the clinicians from the infection team were unable to attend. The aim of the workshop was to provide feedback to the participants from the study and open up the discussion around changes within the department leadership based on the results. As the discussion with the leaders was an important aspect and as those leaders invited also participated, we saw the participation in the workshop as matching its key goal.

The workshop included a brief presentation on the theory and evidence underpinning the role of psychological safety in teams, and of the results from the interviews and questionnaires. ED staff were encouraged to query and discuss any element of the presentation. The discussion points were subsequently used to identify current challenges, and potential modifications in regard to the psychological safety of the team whist engaged in future improvement work. All authors took notes during the workshop on points they found individually relevant for the project.

### Data analysis

#### Interviews

After each interview, interviewers converted their own notes into an electronic format, while revising them for clarity. Each interviewer’s notes were subsequently reviewed by the other interviewer and supplemented or corrected as necessary. Both interviewers agreed on a final version. The audio recordings were consulted to validate the notes in terms of content and completeness.

Interview notes were initially coded deductively and independently by AD, KV, and PD using the ‘antecedents’ and ‘consequences’ of psychological safety from the Edmondson model [9] as thematic categories. This was done electronically in a word processing programme by marking pieces of text with a colour representing a category. Before the rating, it was agreed that in the case of doubt, if a piece of text could not be sorted into an antecedent or consequence category from the model it should be marked for later analysis. This enabled the exploration of additional themes in the material. Double coding of text into more than one category was allowed.

Subsequently, the notes that could not be sorted deductively into any pre-existing category during the first coding step were analysed inductively. PD, KV, and AD independently read the remaining notes and suggested inductive themes covering the content. The themes were identified based on semantic similarities to the notes being coded. The approach taken followed established inductive analytical steps [18]. The whole research team jointly discussed and revised these themes until consensus was reached, and new category headings defined. These headings became the new categories in the revised model. Table 1 demonstrates the sorting procedures with an example, that was translated from Danish to English.

**Table 1:** Example of the coding and summarizing procedures

**Notes Use of practice fields** • I felt safe. The consultant was also there, which was super good. A great help, if there is a need for support • Good idea to try it. It makes a lot of sense for the care process. It’s super to have the overview, to do this yourself and to know that all samples are collected • Several coordinators did not have the test in mind really, but the nurse, who was there during the shift, had an eye on it	**Summarized Notes Use of practice fields** • Having an opportunity to practice helped increase a sense of safety. Plus, having people who are willing to try new ideas, helps to see how those ideas can evolve and can be implemented • People are different in terms of their support for new ideas

#### Questionnaires

Scores from each of the seven statements were totaled. Responses to negatively formulated items were converted. Higher totals indicate more psychological safety. The data from the questionnaires were analyzed with descriptive statistics, showing response distributions.

#### The workshop

After the workshop, the research team collated and reviewed the notes to identify themes and key learning points from workshop. One such theme was ‘problems and solutions’ as identified by the ED staff during the workshop as part of a discussion focusing on ‘Ideas for future improvement work’.

## Results

### Participants

Two nurses and three physicians participated in the pre- and post-interviews. One member of the nursing leadership in the department, one nurse who participated in the test, and one coordinating nurse, who was not part of the test participated in the post interviews. Their professional experience ranged between 7 months and 24 years. Two members of staff participated in the improvement work for one day, one person for two days, one person for four days, and one person for seven days.

### Interviews

The current study is primarily qualitative in nature. Therefore, we report those dynamics that were mentioned by at least one interview participant, but do not report how often a point was mentioned. By conducting the pre- and post-intervention interviews, we did not aim to compare both points in time, nor to use them to ‘evaluate’ the test of work procedures. The reasoning behind this choice was to provide different perspectives of the test; the expectations before the test and the experience of it, afterwards.

In both, the pre- and post-intervention interviews, participants described aspects that could be coded into the antecedent and consequence categories described in the model. Additionally, participants reflected upon the test process, the physical characteristics of the department, how they perceived the tested workflow impacting the quality of care, and how they perceived improvements for the quality and safety of the care for patients. Table 2 provides an overview of the summarized data from the pre-test interviews and Table 3 the summarized data from the post-intervention interviews. The Tables 2 and 3 combine perceptions about psychological safety with descriptions of how it actually played out during the test. In regard to the research question, we consider both perspectives to be of value, and therefore do not distinguish between them, as they offer potential topics for integration into improvement work.

**Table 2:** Interview results from the pre-interviews. Hyphens ( -) indicate that the interviews did not contain points within that respective category

Deductively coded material	
**Team leader** • New suggestions are typically welcome, listened to, decided upon, and tested	**Feedback seeking** -
**Informal group dynamics** • The relationship with colleagues influences the work quality • It is easier to ask for help in face-to-face interactions • One’s own will to engage in the change work depends on how one perceives the enthusiasm for change in the other team members	**Help seeking** • It should be easy and well received to call for help from more experienced colleagues • One needs support in case of errors
**Trust and respect** • Good preparations by other team members can create the trust one needs to feel safe with colleagues • The psychological safety of the person asking for help, depends on the competence of people who are asked for help, and the anticipated likelihood that their advice will actually help • It is important to have faith in colleagues ability to fulfil their responsibilities • Being professional and keeping a respectful tone is important • Good communication and respect for each other are important • Team members are expected to fulfill their responsibilities proactively • Trust that all staff want to find a professional solution for the challenges occurring	**Speaking up** -
**Use of practice fields** -	**Innovation** • Work distribution could be optimized in a way that some tasks could be placed in different roles • There is some resistance to change
**Supportive Organisational Context** -	**Boundary Spanning** -
Inductively coded material beyond the Edmondson model	
**Expectations for the tested way of working** • Hope to be faster in the new work process Positive expectations in terms of quality and speed • The room and equipment have an influence on psychological safety • The physical proximity can improve communication and therefore psychological safety • Challenges anticipated to find the space and time to think • The physical surroundings could be improved **Impact of high workload** • If there are too many patients, one needs to work much faster—that does not feel unsafe, however—psychological safety is not seen as directly influenced by being busy • Psychological safety is endangered because it gets difficult to remember all necessary aspects and one feels stressed • Requires enough personnel and space to make it work • The professional discussions with the physician still function well **The core activity – treating patients safely – is important** • Providing quality care to patients makes healthcare professional feel safe • The focus should always be on the patient • If it is perceived that patient safety suffers, then being busy feels unsafe • Patients had to wait too long **Feeling of being informed** • Details of the new process are unclear • Feeling safe with the project, even though there was little information provided **Change Management** • Too many people involved in the discussions can make things difficult • There should not be too many changes at the same time • It can be unclear, who actually knows which competences and skills, team members actually have

**Table 3:** Interview results from the post-interviews

Deductively coded material	
**Team leader** The team leader can support psychological safety by: • supporting workers in case of challenges • providing sufficient information (keeping in mind that not all information delivered is actually received, understood, accepted, or acted upon) • planning the tasks as well as possible • empowering workers to make the necessary changes to make a change work • communicating own expectations • identifying key change enablers • being present during the change period or by delegating the support of the change clearly	**Feedback seeking** • The close physical proximity and cooperation during the test allowed for an easy dialogue and collaboration • If the people working in this constellation are not collaborating, it can be difficult to seek feedback from each other
**Informal group dynamics** • The close contact to a single physician increased the pressure on the nurse to complete tasks immediately • People react differently to the test process • Close working conditions meant people got to know each other better, but it can also generate feelings of being observed • The contributions of the individuals involved become more visible, one cannot hide behind other group members • Personal differences have stronger impact	**Help seeking** • It was easier to discuss with more experienced colleagues
**Trust and respect** • In close contact, it is important to manage conflicts as early as possible, but this also generates insights into the other person’s perspective, generating more mutual understanding • Process challenges can be interpreted differently by those involved: some see them as rooted in the subject matter, others as rooted in personal issues • The professions are perceived differently: Physicians are considered as more independent and willing to work independently • People vary in how willingly they work with people who are different or unknown • The culture in the department has a big impact on how the test progresses	**Speaking up** • The close contact between the colleagues and the reduced number of patients increased the coupling between the work of those involved and increased the interdependency, requiring more co-ordination and agreements about how to do that • Those involved need to find the matching level of explicitness in the communication
**Use of practice fields** • Having an opportunity to practice helped increase a sense of safety. Plus, having people who are willing to try new ideas, helps to see how those ideas can evolve and can be implemented • People are different in terms of their support for new ideas	**Innovation** • Known procedures felt safe, which makes the work easier • Quality and safety are higher, when the contact between the nurse and the physician is so close • The change is not unequivocally positive, e.g., reduced opportunities for reflection. Telephone-based discussions with colleagues, for example, are more difficult, as they are ideally done away from the patient • The expected effect of more patients being treated was not achieved • Some change ideas are not tested, as those involved do not see them as promising • Not all were convinced of the sense of the trial
**Supportive Organisational Context** • The change (patient pathway) needs to be discussed and agreed upon across the department, and the arrangements put into practice. In particular, co-ordinating nurses have an important role to ensure patients are directed to the new treatment pathway • The co-ordinating nurse is under pressure to send enough patients to the clinical pathway • Physicians and nurses may initially engage with the patient at different points in their care pathway • The dependency between the staff involved increases, e.g., if the one clinician is busy, the other may need to wait. This may contribute to slower task completion • The number of interruptions to clinical work varied throughout the test process • An additional colleague with a flexible role would increase overall task completion • The system was faster for the individual patient, as less patients went through it, but the overall capacity was reduced • The physical resources in terms of room and equipment did not match the tasks. The room was considered too small, the equipment too widely distributed across different locations. This is particularly relevant at the start of test, although adaptations over time reduced this challenge • There is an expectation that some of these challenges could be solved, when the system is established, and the necessary equipment placed in the optimal location • Changes in personnel during the test period required additional time to be spent on introductions and orientation	**Boundary Spanning** • The official task distribution was not questioned, e.g., physicians were not asked to do “nursing” tasks • Adjustments of resources were required, if colleagues had idle time, they offered help in a different task • Only those directly involved in the test mentally engaged in it. This is mainly due to them being so busy that they do not have much capacity to think beyond their immediate jobs • Co-ordination of work is not perceived as an interruption and accepted as a necessary component of collaboration
**Inductively coded material beyond the Edmondson model**	
**Physical characteristics** • The physical context should be optimized in terms of the rooms and the equipment • The room tested was: • Too small • Had no windows • Not temperature regulated • Did not allow access to fresh air to alleviate challenges from smell (especially relevant with the infection patient) • Was perceived as uncomfortable (e.g., because you needed to move equipment to actually exit the room) • Made people feel isolated • Located away from necessary materials • Considered impractical for safe patient care, e.g., patients too close together and confidentially was difficult • Had blue light that over time was perceived as uncomfortable • To consider and discuss patient diagnosis and treatment plan, a separate room (without patients) is necessary • Opinions varied regarding the advantages of such close contact to the patients • For the co-ordinating nurses, the room was far away, making it difficult to keep an overview, e.g., whether the patient was accompanied by a nurse. As an adaptation, often the most unwell patients would be placed closer to the co-ordinating nurse	
**Perceived quality of treatment** • When patients with critical conditions have to wait too long because of delays in the new care pathway, the treating clinicians can feel anxious, due to concerns for the patient’s well-being. This makes made participants feel insecure about the new pathway, as their priority was to treat their patients – consequently, decreasing the perception of psychological safety • The individual perceptions of care quality had a direct impact on how safe participants felt the process was	

### Questionnaire

We invited all eight participants from the infections team to rate the psychological safety in the team during the two-week test period and seven provided their ratings. The overall rating of team psychological safety was 39.8 (Table 4) and therefore can be regarded as very good. In spite of the small sample size, the data show discernable variation in responses. Most noticeably, between statements 5 (*‘It is difficult to ask other members of the team for help* (R)) and 6 *(‘No one on this team would deliberately act in a way that undermines my efforts’*), with ratings of 7.0 and 3.6, respectively.

**Table 4:** Questionnaire results

Statement (rated 1–7, *‘very inaccurate’* to *‘very accurate’*)	1	2	3	4	5	6	7	Mean (*n* = 7)
1. If you make a mistake in this team, it is often held against you (R)	0	0	0	0	0	2	5	**6,7**
2. Members of this team are able to bring up problems and tough issues	1	0	0	0	1	1	4	**5,7**
3. People on this team sometimes reject others for being different (R)	0	0	0	1	1	2	3	**6,0**
4. It is safe to take a risk on this team	0	1	0	2	1	2	1	**4,9**
5. It is difficult to ask other members of this team for help (R)	0	0	0	0	0	0	7	**7,0**
6. No one on this team would deliberately act in a way that undermines my efforts	2	2	0	0	1	0	2	**3,6**
7. Working with members of this team, my unique skills and talents are valued and utilised	1	0	0	0	0	2	4	**5,9**
**Total team score (Mean)**	**39,8 (5,7)**							

^a^Ratings for statements with (R) are reversed

### Workshop

Participants saw the presentation of the interview and questionnaire results as fitting with their view of the team. They made positive remarks about the value of using the Edmondson framework to understand their own experiences. Participants also described the results as relevant for their work to support future testing and learning towards ongoing improvement work and ‘day-to-day’ delivery of safe and reliable care.

The discussion around challenges identified from the qualitative and quantitative data, along with potential change ideas to help test solutions yielded six areas for change focusing on communication, collaboration, and technical issues. We agreed with participants at the workshop that no details of the discussion would be published.

### Summary

All antecedents and consequences could be populated with interview material. As such, the model provides a useful framework to interpret our data. The independent coding showed good agreement in the majority of cases, even though formal agreement calculations were not used due to small sample size. Any disagreements in coding could be resolved in discussions within the research team. Similarly, when these data were presented in the workshop to ED staff, participants regarded the coding as valid, useful and accurately reflected their view of the team.

### Revised model of psychological safety

**Fig. 3:**
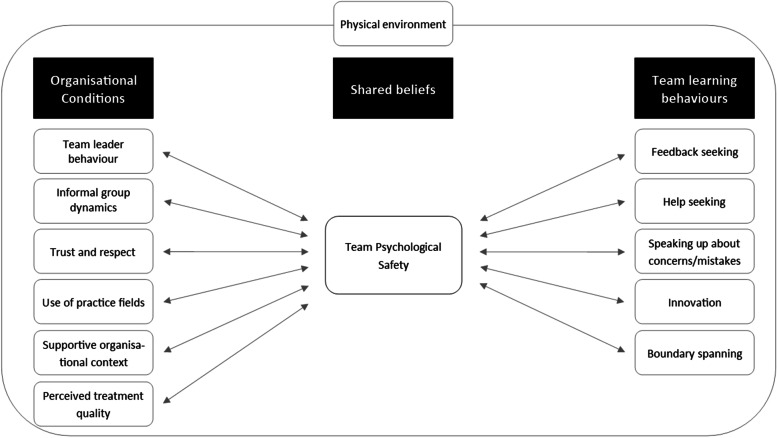
Modified model of psychological safety

Based on the results from the different data sources, we suggest that when applied to improvement work, it may be appropriate to modify Edmondson’s model in three ways (Fig. 3):

1. ‘Physical environment’ We have added this element to the model as a background within which all other elements exist. The interview and workshop data refer to the importance of the physical space in which participants were engaged in improvement work, specifically factors such as sufficient space to undertake the clinical processes appropriately or proximity to essential equipment.
2. ‘Perception of treatment quality’ We believe it is appropriate to add this element as an antecedent because participants described that their assessment of the quality of care and treatment patients received influenced how psychologically safe they themselves felt to engage in testing new ways of working. Participants’ perceived psychological safety decreased, where they saw the care for their patients at risk.
3. ‘Bi-directional arrows’ We believe the data from this study support a modification of the model, from unidirectional arrows to bi-directional arrows. This would therefore articulate that perceptions and experience of the ‘outcome factors’ (learning behaviours), would in turn provide confirmation that psychological safety in the team is high and thus reassure members of the team that the antecedent factors are relevant and worth developing. Bi-directional arrows would more accurately reflect the dynamic nature of group interaction and would also allow for negative feedback loops, i.e., if examples of learning behaviours are not witnessed in a group, this could undermine and reduce faith in the antecedent factors and overall psychological safety.

## Discussion

### Main findings

We investigated whether the Edmondson model of psychological safety is a useful framework to help understand staff experiences of engaging in improvement work in an emergency department (ED) in Denmark. We collected data with semi-structured interviews, a questionnaire, and via a workshop. We found that the model was applicable in this context but did not comprise all the points identified as important by the study participants. We have suggested revisions to the model based on our results.

Our sample is small and therefore does not warrant the revision of a well-established model based on these empirical findings alone. We therefore supplement our suggestions for revisions with theoretical considerations. In the following section, we unfold our theoretical reasoning to help understand better our suggestions to modify the model.

### Physical characteristics

The physical characteristics of the space for the improvement work was a prominent theme in the data. Edmondson [9] only briefly touches upon the issue of physical space in terms of being a component of ‘Organisational Structure’ (an antecedent). However, research has long identified the physical environment as having a significant impact on human experiences in general [19], and specific issues such as safety and performance in healthcare [20–22], as well as patient satisfaction [23]. More recently, there has been a focus on the psychologically safe environment specific to learning during simulation [24]. Our data indicate that physical characteristics play a significant role in the perception of psychological safety for healthcare professionals. It reflects the assertion that the interrelationships between humans, the tools they use, and the environment in which they live and work [25] needs to be jointly analysed and optimized in order to make changes or progress [26].

When testing new work procedures, both the functional and psychological value of physical spaces should be accounted for. The functional value describes whether the space supports task management. The psychological value describes how people think about and experience a space, specifically, what kind of value they assign to the space and what they extrapolate from the experienced value of the space about their own standing (value) in a team or organisation. This is relevant for planning, conducting, and interpreting tests or changes of new processes.

Changing the physical aspects of work, involves learning to navigate a new physical layout; a process which may take time. Any test of change would therefore need to acknowledge and account for the rate of progress in gaining knowledge and skills (learning curves). This is equally true for changes in equipment or layout within a specific location. This would mean that a test of a new work process that is terminated before the users have developed some familiarity with the new layout might not allow for valid and reliable conclusions.

Based on these theoretical considerations, we therefore propose that the physical characteristics of improvement work are an important factor influencing team psychological safety, especially during activities which may be new or uncertain, as is typically the case in improvement work.

### Perception of treatment quality and safety

The interview data concerning ‘the perception of treatment quality and safety’ relate closely to two issues. Firstly, an individual’s ‘expectations’ about the new way of working, and the subsequent influence this has on psychological safety. Secondly, an individual’s motivation. Although not included in Edmondson’s model, the issues of expectations and motivation and how they relate to psychological safety have been covered previously in the literature [9]. For example, Edgar Schein argued that psychological safety helps people overcome the defensiveness, or learning anxiety, that occurs when they are presented with data that contradict their expectations. [27] With psychological safety, he reasoned, individuals are free to focus on collective goals and problem prevention rather than on self-protection. As we propose later (see: ‘bidirectional relationships’), the same could also be true in reverse, i.e., an individual’s positive expectations of the changes being tested could influence their sense of psychological safety, especially if their expectations are based on prior experience or best practice from a different context.

It is possible to approach the issue of motivation and psychological safety from a number of perspectives. In their systematic review, Newman and colleagues [28] suggest ‘Conservation of Resources (COR) theory’ [29, 30] as a useful approach to explain how access to resources in the work environment motivates employees to invest their own resources at work to help others, and to stimulate learning, growth and development. COR theory explains how individuals and groups strive to obtain, retain, protect, and invest in resources to sustain their resource pool [29, 30]. Resources are defined as objects (e.g., work tools, house), personal characteristics (e.g., optimism, self-efficacy), conditions (e.g., seniority, tenure), and energies (e.g., knowledge, time) valued by individuals, who either receive or provide these resources. According to COR theory, individuals have limited resources and are highly motivated to protect them [30]. Proponents of the theory argue that to gain better insight into stressful situations, such as testing new ways of working, it is best to focus on the resources that are available to deal with the stressful situation rather than the situation itself [31].

The basic premise of the resource investment principle which is a key component of COR theory is that individuals invest in resources to get more resources, to protect against the possible loss of resources, and to recover from losses when they occur [32]. This possibility of loss is implicit during improvement work and when engaging in learning behaviours such as speaking up or offering ideas. Even when a person offers constructive ideas, there is still an element of risk as speaking up itself suggests a challenge to the existing structures and authorities [33]. Therefore, investing in supportive work relationships during improvement work is important because they facilitate the preservation, sustenance, or depletion of a person’s resources [30] and therefore psychological safety.

Conservation of Resources Theory [30] can therefore be seen as a way to explain how individuals generate psychological safety by proactively accessing, reserving, and recovering resources in the work environment. This is a relatively novel perspective. Previous antecedents to psychological safety have ignored the role of individuals in actively adapting to the environment and only emphasized the impact of external factors such as supportive leadership behaviours, relationship networks, and team characteristics [34] on psychological safety.

The ‘Perception of treatment quality and safety’ factor emphasizes the importance of ‘motivation’ to engage in the improvement work. If staff perceive that the proposed changes may impact negatively on the quality or safety of the care the patient receives, the staff members’ reason for doing the job is challenged. This was described by participants in this study as having an impact on their psychological safety and helped to establish a link between the actions of healthcare professionals and the outcome of these actions. In our experience from education, training, and improvement projects in healthcare, it is not always easy to make this link visible. Often the focus is either on the processes (e.g., finding diagnoses, collaborating in a team, leading, following, etc.) or the outcomes. Less on the connection between both. Our interview data highlight this link, specifically with the notion of decreased psychological safety, if new ways of organizing the work compromise the motive of staff.

Therefore, even if many of the other antecedent factors are robust, such as ‘supportive and inclusive leadership’, high levels of interprofessional ‘trust and respect’, etc. these favourable contextual factors may be insufficient to foster high levels of psychological safety if the staff perceive treatment quality or patient safety as being compromised. Improvement work in healthcare typically involves identifying a clear and compelling shared goal. People are more likely to offer ideas, ask for help, and seek or provide feedback if they believe that their effort makes a difference in achieving an outcome that they care about [10].

These findings would suggest that during the establishment and testing of improvement work, it is important to consider staff expectations and motives as critical antecedents which can impact on psychological safety and subsequent learning behaviours.

### Bidirectional arrows

We suggest changing the arrows in the model from uni- to bidirectional arrows. Whilst the evidence for the direction from antecedents to consequences is strong, and these feel instinctively correct, we believe that, in this context at least, the experience of witnessing learning behaviour had a confirmatory impact on the psychological safety of members of the team, which should be expressed graphically by the arrows pointing in both directions.

By utilizing bidirectional arrows in the model, while keeping psychological safety as a mediator of the connections, we believe this better acknowledges the complex nature of engaging in improvement work in healthcare settings and the information flow between team members. While this may make the model less compelling at first glance, we believe that it facilitates consideration of psychological safety as a more dynamic framework by acknowledging the complexity of the situation.

This reciprocal relationship is common throughout the literature in this field [35, 36]. As highlighted early [37] these dynamic reciprocal relationships better reflect a model of reciprocal determinism [38]. Bandura states that reciprocity does not mean that the different sources of influence are of equal strength, neither do the reciprocal influences occur simultaneously [38]. Rather, it takes time for a causal factor to exert its influence and to activate reciprocal influences. This bidirectionality of influence means that people are both products and producers of their environments. We believe this is equally true of psychological safety and therefore propose the use of bidirectional arrows in the model.

### Limitations

This study was exploratory, and its findings are limited. The interview and questionnaire samples are small and might therefore not warrant the changes suggested alone. Whilst acknowledging this, we see a fit between the empirical findings and theory relevant to these issues. Therefore, we believe there is sufficient grounds to propose our modifications for discussion and further theoretical and empirical testing.

In the post-intervention interviews, participants covered more of the different elements in the model. This may be due to several reasons, including prior discussions about similar issues, the fact that we interviewed more people after the test, and that we might have influenced the participants. However, our interpretation is that participants cannot fully predict all the implications of the improvement work and that there might well be a difference in the assumptions, expectations, and wishes before a test and the concrete experiences after the test. This emphasizes the need for research in improvement work to explore the perceptions of those involved as one very valuable source of data amongst.

In the questionnaire, we suspect a translation problem from English to Danish with the item 4 (“It is safe to take a risk in this group”). The Danish translation of this item suggests more to take risk regarding patient treatment and not so much the interactional risks. This issue has been highlighted elsewhere [14] and may help to explain the variation in data relating to this question.

We worked with paraphrased core points from the interviews and did not transcribe the interviews completely. We might have misunderstood some points and noted others in a way that does not fully reflect the meaning of the points made by interviewees, even though we took care to ask participant to comment on our notes.

We consider the leadership and professionals in the department as interested in and supportive of both engaging in improvement work and the role of psychological safety in teams. As such, our sample may not be representative of the broader healthcare professional workforce. Leaders play a pivotal role in fostering psychological safety. In our study, the leadership team initiated and supported the improvement work, as well as the focus on the psychological safety aspect of the work for team members participating in the improvement work. We believe this created a clear and consistent message for the clinicians participating in the study, namely that leadership supported them and had a genuine interest in and willingness to support the improvement work. This message was reiterated by the leadership attendance at the workshop and can be seen in the results from the questionnaire. This support and interest may also have positively influenced the findings from this study.

Finally, the findings we present stem from an emergency department with its own culture, traditions, and norms. Given the unique role and function ED’s have within the healthcare system we are unsure how generalizable our findings are for care settings that do not share these characteristics.

### Reflexivity

Any research is impacted by those who carry out the studies, who collect, analyse, interpret, and present data. This is especially true for qualitative studies. We already discussed the limitations we see in the study. In this section, we reflect upon the relationship between us as researchers, the study participants, and the context in which we conducted the study.

Our research group consists of three psychologists and a nurse, who is also responsible for patient safety in the department in which the study was conducted. At the time of the study, two of us (ST and PD), are employed outside of the hospital in which the study took place, one (KV) worked in a different department of the same hospital, while one was employed in the department (AD). While discussing the results of our study, we tried to draw on our different professional background to critically evaluate them. We discussed all points until agreement.

When revising such an established model as the Edmondson model based on a small study, we were unsure at times, whether our data warrants our suggestions. After many discussions we decided that the points made by our participants and our own understanding of the role of physical places, of complexity, and the influence of perceptions on actions, were strong enough to form the suggestions we formulated.

Our participants might have felt “tested” regarding their contribution in the actual test of the infection pathway. We tried to explain our different perspective for this study but cannot exclude that participants might have felt the need to present their role in a “good light”, potentially downplaying related challenges.

The context in which we conducted the study was close to the actual test. That means that we captured some fresh impressions, which might be further qualified and refined over time and it would have been nice to do a follow up study, which was not possible in the context of our project.

## Conclusion

We conclude that the Edmondson model of psychological safety was applicable to describe many of the dynamics experienced by staff engaged in testing new work processes in an emergency department, but that the model did not cover all aspects that were seen as relevant by study participants. We have suggested modifications to the model in three aspects. Specifically, we added the physical characteristics as a background for the whole model, an antecedent factor that focusses on the core activity of treating patients and relates to staff expectations and motivation. And finally, we suggest the connections in the model should be bi-directional to better reflect the underlying complexity and dynamism of psychological safety in teams engaged in improvement work. In conclusion, we believe the revised model of psychological safety is helpful to understand better the dynamic interplay between contextual factors and subsequent learning behaviours for staff engaged in testing new ways of working in acute healthcare conditions.

## Supplementary Information

**Additional file 1.**

## Data Availability

The datasets generated and analysed during the current study are not publicly available per agreement with the leadership of the participating department but are available from the corresponding author on reasonable request.

## Abbreviations

ED: Emergency Department(s)
VUCA: Volatile, uncertain, complex, ambiguous
AD: Anne Eva Dalgaard
KV: Kirsten Varming
PD: Peter Dieckmann
ST: Simon Tulloch
R: Reverted questionnaire item
COR theory: Conversation of resources theory
