# Supplementary material for: Psychological safety during the test of new work processes in an emergency department
Source: BMC Health Serv Res. 2022 Mar 5;22:307. doi: 10.1186/s12913-022-07687-y (PMC8898467; doi:10.1186/s12913-022-07687-y)
Supplement: Supplementary file 1 — Additional file 1. [file 12913_2022_7687_MOESM1_ESM.docx]

Thank you for agreeing to participate in this interview. As discussed, all information is confidential and will only be seen by members of the research team.

| **Pre-intervention (test of the infection pathway)** | |
| --- | --- |
| Title: physician, nurse, coordinator, other (please specify)  How long since you graduated?  How long have you worked in the emergency department? | Job title:  Graduated:  Under 6 month/ months to 2 years/ above 2 years |
| 1. What does psychological safety mean for your work? |  |
| 1. How do you try to create psychological safety in multi-professional work?  - What can hinder you in this process? |  |
| 1. What do you do when you are concerned about a patient?  - Do you feel psychologically safe to ask for help / seek advice? - Are you concerned about disturbing your colleagues? - Do you manage the situation on your own? |  |
| 1. When you think about the new work process you are about to test - how psychologically safe do you feel? |  |
| 1. Do the physical characteristics of the department have an influence on your experience of psychological safety in relation to multi-professional collaboration?  - Yes: please describe in what way - No: please describe why not |  |
| 1. What thoughts do you have about organising the work in the emergency department in the form of specialized pathways? |  |
| 1. When you think back to last week – how psychologically safe were you in regard to patient treatment?  - Was there anything that made you insecure? If yes – why was that? |  |
| 1. Can you remember some examples for positive multi-professional collaboration from last week?  - Which factors, do you think, contributed to this positive example? |  |
| 1. Which changes in work processes do you think will result in a work environment with better care and treatment? |  |
| 1. When a co-worker in the department offers an idea for new ways of working, what would typically happen? |  |
| 1. Is there anything that you think is important about the new work process that we did not ask about? If yes – what was it? |  |

| **Post intervention (test of the infection pathway)** | |
| --- | --- |
| Title: physician, nurse, coordinator, other (please specify) |  |
| 1. How many days did you participate in the new infection pathway process? |  |
| 1. How many colleagues did you work with during the process? |  |
| When you think of the new work process:   1. How psychologically safe did you feel during the test? E.g., compared to the usual work process. 2. Were there differences in the different groups in which you tested the infection pathway? (NB: we do not share data with your colleagues) |  |
| 1. Did the physical aspects of the new work process have any impact on your psychological safety in regard to multi-professional collaboration? |  |
| 1. How do you see the new work pathways working in the emergency department? 2. What was the most important learning point for your from testing the new pathway process? |  |
| 1. When you think about the two weeks of testing:  - How psychologically safe did you feel in regard to your contribution to the team? - What do you think about the safety and quality of patient treatment? - Was there something that made you feel less secure? If yes, please explain why. |  |
| 1. Can you remember examples of good collaboration during the course test period?  - What factors contributed to this good collaboration? |  |
| 1. Do you have any recommendations on how the new pathway process could be improved or optimized? |  |
| 1. Is there something we did not ask and that you think is important to mention? |  |

Thank you for your participation in this interview. If you have any questions about this interview or any part of the research, you are welcome to contact either Peter Dieckmann ([gerhard.peter.dieckmann@regionh.dk](mailto:gerhard.peter.dieckmann@regionh.dk)) or Anne Eva Dalgaard ([anne.eva.dalgaard.01@regionh.dk](mailto:anne.eva.dalgaard.01@regionh.dk))
